# Tender Cutaneous Neoplasms: Case Reports of Patients With a Symptomatic Dermatofibroma and a New Acrostic for Painful Tumors of the Skin

**DOI:** 10.7759/cureus.29713

**Published:** 2022-09-28

**Authors:** Philip R Cohen

**Affiliations:** 1 Dermatology, University of California, Davis Medical Center, Sacramento, USA

**Keywords:** tumor, tender, skin, painful, neoplasm, mnemonic, dermatofibroma, dermal, cutaneous, acronym

## Abstract

The most common tender cutaneous neoplasm is a dermatofibroma. The characteristics of three women (35 to 42 years old) with painful dermatofibromas are described. One woman was receiving immunosuppressive therapy for the past decade following a liver transplant; the other women were healthy. The dermatofibroma was located on the buttock, shoulder, and arm, respectively; tumor-related pain had been present for several months to at least a year. The dermal nodules ranged in diameter from 5 millimeters to 12 millimeters and were either flesh-colored to slightly red or brown or tan; one tumor was surrounded by a hyperpigmented patch. Microscopic examination showed the following dermatofibroma variants: classic (fibrocollagenous) in two women and histiocytic in one woman. All the women experienced resolution, without recurrence, of pain following the punch biopsy that only removed the majority (but not all) of the dermatofibroma. Tender cutaneous neoplasms include not only dermatofibromas and other fibrous lesions, but also adipose, bone, calcium, cartilage, eccrine, infiltrative, lymphoproliferative, muscle, neural, and vascular tumors. Acronyms and acrostics are mnemonic devices that have been used by clinicians to aid in recalling the diagnoses associated with painful skin tumors. When there were only 11 or less number of pain-related cutaneous conditions, shorter acronyms associated with either a woman’s name, a country or an egg were used. A unique acrostic inspired by Charlotte’s Web, a children’s book by E. B. White, was created when the differential diagnosis of tender cutaneous neoplasms expanded to 25 tumors. The sequential discovery of additional tender skin lesions resulted in two subsequent minor revisions to the original, hog-related, mnemonic. Herein, a new acrostic -- that is not only able to incorporate the inspiration from Charlotte’s Web, but also includes cutaneous lymphoma and a final category of “everything else” in order to maintain the future integrity of mnemonic -- for the painful tumors of the skin is proposed: HOG FLED PEN AND GETS CALM LIFE BACK (hidradenoma, osteoma cutis, glomus tumor, fibromyxoma [superficial acral], leiomyosarcoma [cutaneous], eccrine angiomatous hamartoma, Dercum’s disease, piezogenic pedal papule, eccrine spiradenoma, neurilemmoma, angiolipoma, neuroma, dermatofibroma, granular cell tumor, endometriosis [cutaneous], thrombus [cutaneous organizing], scar, calcinosis cutis, angioendotheliomatosis [reactive], leiomyoma, metastases [cutaneous], lymphoma [cutaneous], intravenous lobular capillary hemangioma, foreign body [and foreign body reaction], everything else, blue rubber bled nevus, angioma [tufted], chondrodermatitis nodularis helicis, and keloid).

## Introduction

Painful tumors of the skin include a variety of benign and malignant neoplasms. They include adipose, bone, calcium, cartilage, eccrine, fibrous, infiltrative, lymphoproliferative, muscle, neural, and vascular tumors. Mnemonic devices -- such as acronyms and acrostics -- have been created to aid clinicians in remembering the differential diagnosis of painful skin tumors [1-8].

Dermatofibroma is a common benign cutaneous neoplasm. A solitary tumor may be the consequence of an insect bite at the site of the lesion whereas some of the individuals with multiple lesions may have an acquired condition that alters their immunity or be a recipient of treatment with an immunosuppressive agent or both. Most dermatofibromas present as asymptomatic dermal papules or nodules; however, albeit less frequently, the tumor may be painful [9-15].

The clinical features of three women, each of whom developed a painful dermatofibroma, are described. In addition, the characteristics of tender dermatofibromas that have been reported in other individuals are summarized. Also, previous and current mnemonics for tender cutaneous lesions are reviewed and a new acrostic for painful tumors of the skin is proposed.

## Case presentation

Case one

A 42-year-old Philippine woman presented with a tender lesion on her left buttock of approximately one-year duration. It had begun as a small dark spot and had continued to slowly enlarge. She had no previous skin cancers or dermatologic conditions.

**Figure 1:**
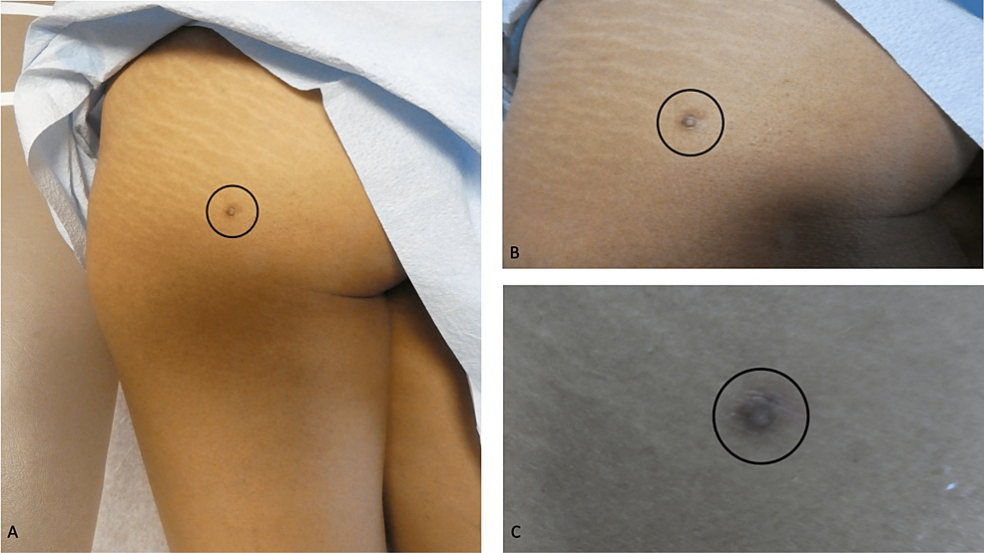
Morphologic features of a painful dermatofibroma on the buttock Distant (A) and closer (B, C) views of a painful dermatofibroma (within the black oval) on the lateral and inferior area of her left buttock of a 42-year-old Philippine woman. The tender tumor had been present for approximately 12 months and appears as a hyperpigmented 3 x 2-centimeter patch surrounding a protuberant brown 1.2 x 1.2-centimeter dermal nodule.

A complete cutaneous examination was done. A protuberant, painful, brown 12 x 12-millimeter dermal nodule surrounded by a hyperpigmented 3 x 2-centimeter patch was observed on the lateral and inferior area of her left buttock (Figures 1A-1C). A three-millimeter biopsy from the nodular portion of the skin lesion, using the punch technique, was performed.

**Figure 2:**
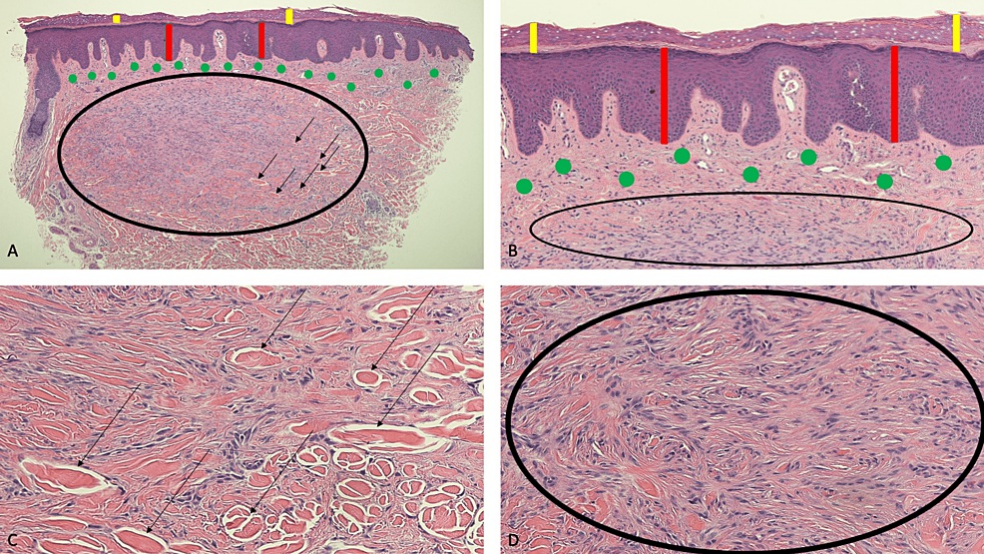
Pathologic features of a tender dermatofibroma in a 42-year-old Philippine woman Distant (A) and closer (B-D) views of microscopic findings of painful dermatofibroma (of the classic or fibrocollagenous variant). The epidermis (A, B) shows not only increased thickening of the stratum corneum without retention of nuclei within the cells (orthokeratosis, demonstrated by the yellow vertical bars), but also thickening of the other layers (acanthosis, demonstrated by the red vertical bars). Beneath the epidermis, in the dermis above the tumor, there is an area of normal-appearing papillary dermis (solid green circles) referred to as a Grenz zone (A, B). The dermal tumor (within the black ovals) appears as a whorled arrangement of fibroblasts and collagen (A, B, D); it extends from the papillary dermis into the reticular dermis. At the periphery of the dermatofibroma (A, C), the collagen bundles are compressed and have a keloid-like appearance (black arrows) (Hematoxylin and eosin: A, x4; B, x10; C, x20; D, x20).

Microscopic evaluation of the tissue specimen showed a benign spindle cell tumor, consisting of a predominance of collagen and fibroblasts in a whorled arrangement, extending from the deeper portion of the papillary dermis into the reticular dermis and extending to the lateral margin of the specimen; compressed collagen bundles, having a keloidal appearance, were noted at the periphery of the neoplasm. A grenz zone of the normal-appearing papillary dermis was noted between the underlying dermal tumor and the overlying epidermis. The epidermis showed acanthosis and orthokeratosis (Figures 2A-2D).

The correlation of the clinical presentation and the pathology findings established the diagnosis of a painful dermatofibroma of the classic variant; this tumor has also been referred to as a fibrocollagenous dermatofibroma. Most of the benign dermal neoplasm had been removed during the biopsy; therefore, the residual tumor and surrounding hyperpigmentation were observed. The dermatofibroma-associated pain completely resolved after the biopsy site had healed and there has been no recurrence of pain at the site.

Case two

A 37-year-old Hispanic woman presented with a painful lesion on her left shoulder of several months duration. It had begun as a small tan patch that had grown to its current size and become painful. She also had warts on her feet.

Her medical history was significant for a liver transplant 10 years earlier and episodic migraine headaches. Her transplant-related medications included 1,000 milligrams daily of mycophenolate mofetil and two milligrams twice daily of tacrolimus. She would take 50 milligrams of sumatriptan when she began to develop a headache. Her plantar warts were being treated topically by applying 40% salicylic acid pads daily.

**Figure 3:**
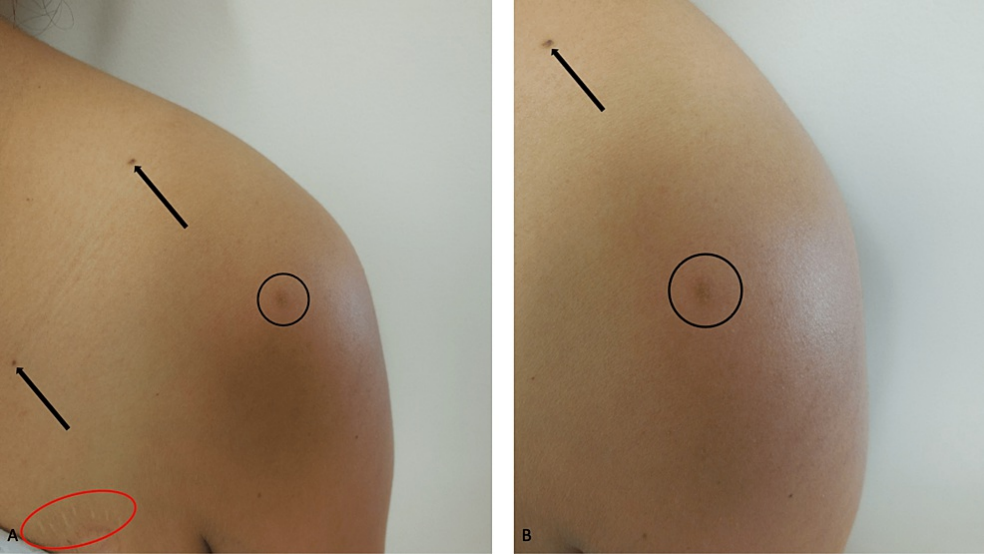
Clinical presentation of a tender dermatofibroma on the shoulder Distant (A) and closer (B) views of a painful dermatofibroma of several months duration on the left shoulder (proximal to the arm) of a 37-year-old Hispanic woman who was a liver transplant recipient ten years earlier and has been maintained on daily, transplant-related immunosuppressive therapy consisting of mycophenolate mofetil and tacrolimus. The tender dermatofibroma of the classic or fibrocollagenous variant (A, B) appears as a tan 5 x 5-millimeter, slightly raised, dermal nodule (within the black circle). In addition, benign-appearing pigmented lesions, clinically consistent with junctional nevi (black arrows) are present on the left chest (A) and left shoulder (A, B). Also, on the left chest above the breast (within the red oval), a healed scar can be noted (A).

A complete cutaneous examination was done. A slightly raised, painful, tan 5 x 5-millimeter dermal nodule was noted on the left shoulder proximal to her arm (Figures 3A, 3B); dimpling of the skin was observed when the corresponding area was pinched between the clinician’s thumb and index finger. A 3-millimeter punch biopsy, from the center of the tender skin lesion, was performed.

Two benign-appearing brown patches were noted on the left shoulder (superior and medial to the painful dermal lesion) and left chest; clinically the lesions were consistent with junctional nevi. In addition, there was a healed scar on her left chest above the breast. Also, several verrucous papules were present on the great toe and plantar surface of her left foot; these were plantar verruca.

Microscopic evaluation of the tissue specimen from the left shoulder showed a non-circumscribed, benign-appearing, fibrocellular tumor in the reticular dermis. The tumor consisted of intertwining bands of collagen interspersed with predominantly fibroblasts and occasional histiocytes. The epidermis was acanthotic and its basal layer was hyperpigmented.

The correlation of the clinical presentation and the pathology findings established the diagnosis of a painful dermatofibroma of the classic variant. Almost all the dermal tumors had been removed during the biopsy and the site of the residual dermatofibroma was clinically observed. The pain previously associated with the dermatofibroma did not persist after the biopsy and did not recur.

Case three

A 35-year-old Caucasian woman presented with a painful mass on her left upper extremity of at least 12 months duration. It had begun as a small bump and became tender as it grew. The patient was healthy and had no other skin conditions.

**Figure 4:**
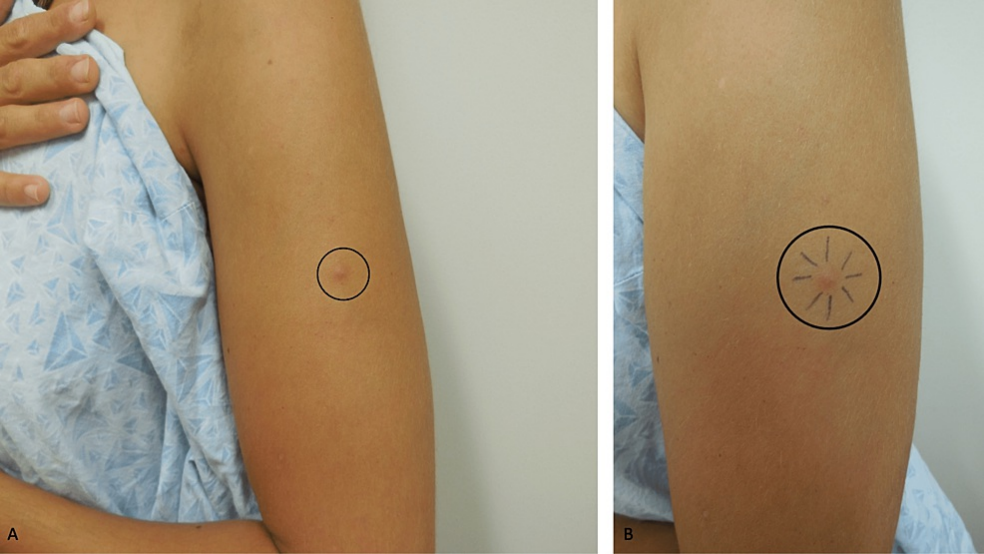
A tender dermatofibroma presenting as a painful dermal nodule on the arm Distant (A) and closer (B) views of the painful histiocytic dermatofibroma of at least one-year duration appearing as a flesh-colored to slightly red 8 x 8-millimeter prominent dermal nodule (within the black circle) on the left extensor mid-arm of a 35-year-old Caucasian woman. A pen with black ink (B) was used to draw eight lines toward the lesion until the dermal component was contacted in order to approximate the diameter of the dermatofibroma.

A complete cutaneous examination was done. A prominent, tender, flesh-colored to slightly red 8 x 8-millimeter dermal nodule was observed on the left extensor mid-arm (Figures 4A, 4B). A three-millimeter biopsy from the center of the nodule, using the punch technique, was performed.

Microscopic evaluation of the tissue specimen showed a dermal tumor, consisting of numerous histiocytes and some fibroblasts that extended to the lateral margin of the specimen; within the benign neoplasm, there was hemosiderin and phagocytosis of lipid by the histiocytes. There was acanthosis of the overlying epidermis.

The correlation between the clinical presentation and the pathology findings established the diagnosis of a painful dermatofibroma of the histiocytic variant. The biopsy site healed; the site and residual tumor were clinically observed. The dermatofibroma-associated pain resolved without recurrence.

## Discussion

Several neoplasms are included in the differential diagnosis of painful dermal tumors (Table 1) [1-6]. The tender skin lesion typically presents as a cutaneous nodule that is either flesh-colored or maybe blue, brown, purple, or red. Microscopic evaluation of a tissue specimen -- obtained during biopsy or excision of the lesion -- is usually necessary to establish the diagnosis [1-6].

**Table 1:** Acronyms and acrostics for painful tumors of the skin Abbreviations: CR, current report; Ref, reference. ^a^Aids that can be used to assist a person in remembering something are referred to as mnemonic devices. Acronyms and acrostics are mnemonic devices. An acronym is usually one word, but can be several words, made up of the first letters of other words that you want to remember. An acrostic, similar to an acronym, uses the first letter of each item you want to remember to create a memorable sentence or phrase. ^b^Prior to these acronyms, a table of painful tumors of the skin was published in 1985 and included leiomyomas, glomus tumor, eccrine spiradenoma, neuroma, and neurilemmoma [1]. ^c^Based on topic-related literature, these acronyms had not been published prior to 1986 [2].

Acronym or acrostic^a^	Painful tumors of the skin	Year	Ref
GLENDA^b^	Glomus tumor, leiomyoma, eccrine spiradenoma, neuroma and neurilemmoma, dermatofibroma, and angiolipoma	1986^c^	[2]
ENGLAND^b^	Eccrine spiradenoma, neuroma, glomus tumor, leiomyoma, angiolipoma, neurilemmoma, and dermatofibroma	1986^c^	[2]
LEND AN EGG	Leiomyoma, eccrine spiradenoma, neuroma, dermatofibroma, angiolipoma, neurilemmoma, endometrioma, glomus tumor, and granular cell tumor	1993	[2]
BLEND TAN EGG	Blue rubber bleb nevus, leiomyoma, eccrine spiradenoma, neuroma, dermatofibroma, tufted angioma, angiolipoma, neurilemmoma, endometrioma, granular cell tumor, and glomus tumor	2019	[3]
CALM HOG FLED PEN AND GETS BACK	Calcinosis cutis, angioendotheliomatosis (reactive), leiomyoma, metastases (cutaneous), hidradenoma (also referred to as clear cell acrospiroma, clear cell hidradenoma, eccrine acrospiroma, nodular hidradenoma, and solid-cystic hidradenoma), osteoma cutis, glomus tumor, fibromyxoma (digital, which is also referred to as a superficial acral fibromyxoma), leiomyosarcoma (cutaneous and originating in either the dermis or subcutaneous tissue), eccrine angiomatous hamartoma, Dercum’s disease, piezogenic pedal papule, eccrine spiradenoma, neurilemmoma, angiolipoma, neuroma, dermatofibroma, granular cell tumor, endometriosis (cutaneous), thrombus (cutaneous organizing thrombus which was originally referred to as a capillary aneurysm of the skin), scar, blue rubber bled nevus, angioma (tufted variant), chondrodermatitis nodularis helicis, and keloid	2019	[4]
CALM HOGS FLED PENS AND GET BACK	Calcinosis cutis, angioendotheliomatosis (reactive), leiomyoma, metastases (cutaneous), hidradenoma, osteoma cutis, glomus tumor, scar, fibromyxoma (superficial acral), leiomyosarcoma (cutaneous), eccrine angiomatous hamartoma, Dercum’s disease, piezogenic pedal papule, eccrine spiradenoma, neurilemmoma, something else (including foreign body such as solder and a reaction to the foreign body), angiolipoma, neuroma, dermatofibroma, granular cell tumor, endometriosis (cutaneous), thrombus (cutaneous organizing), blue rubber bled nevus, angioma (tufted), chondrodermatitis nodularis helicis, and keloid	2020	[5]
IF HOGS FLED PEN, CALM AND GET BACK	Intravenous lobular capillary hemangioma, foreign body (reaction), hidradenoma, osteoma cutis, glomus tumor, scar, fibromyxoma, leiomyosarcoma, eccrine angiomatous hamartoma, Dercum’s disease (adiposis dolorosa), piezogenic pedal papule, eccrine spiradenoma, neurilemmoma (schwannoma), calcinosis cutis, angioendotheliomatosis, leiomyoma, metastases, angiolipoma, neuroma, dermatofibroma, granular cell tumor, endometriosis, thrombus, blue rubber bled nevus, angioma, chondrodermatitis nodularis helicis, and keloid	2022	[6]
HOG FLED PEN AND GETS CALM LIFE BACK	Hidradenoma, osteoma cutis, glomus tumor, fibromyxoma (superficial acral), leiomyosarcoma (cutaneous), eccrine angiomatous hamartoma, Dercum’s disease, piezogenic pedal papule, eccrine spiradenoma, neurilemmoma, angiolipoma, neuroma, dermatofibroma, granular cell tumor, endometriosis (cutaneous), thrombus (cutaneous organizing), scar, calcinosis cutis, angioendotheliomatosis (reactive), leiomyoma, metastases (cutaneous), lymphoma (cutaneous), intravenous lobular capillary hemangioma, foreign body (and foreign body reaction), everything else, blue rubber bled nevus, angioma (tufted), chondrodermatitis nodularis helicis, and keloid	2022	CR

Dermatofibroma, also referred to as a fibrous histiocytoma, maybe a painful dermal tumor. However, this benign neoplasm was not included in the manuscript that summarized tender cutaneous lesions by Thompson in 1985 (Table 2) [1-8]. Subsequently, dermatofibroma was incorporated into the early acronyms (such as GLENDA and ENGLAND) for painful tumors of the skin (Table 1) [1-6]. Indeed, Naversen et al. considered dermatofibroma to be the most common of the painful lesions when they included the tumor in their LEND AN EGG acronym [2].

**Table 2:** Landmark contributions in the development of mnemonics for painful tumors of the skin Abbreviations: CR, current report; Ref, reference. ^a^Twelve years earlier (in 2005), one of the coauthors (Dr. Ramesh M. Bhat) along with two additional investigators described a 31-year-old man with multiple painful acquired tufted angiomas of 1.5 years duration on his chest [7].

Author	Year	Contribution	Ref
Thompson	1985	The author reported a 24-year-old man with biopsy-confirmed multiple painful pilar leiomyoma on his left chest from the mid-sternum to the areola; the pain was relieved with oral nifedipine (10 mg three or four times daily). A table summarizing painful tumors of the skin (leiomyomas, glomus tumor, eccrine spiradenoma, neuroma, and neurilemmoma) was included in the paper.	[1]
Naversen, et al.	1993	The authors described a 19-year-old woman with a biopsy-confirmed granular cell tumor on her right chest that was painful and tender; this lesion was not included in the prior acronyms, such as GLENDA and ENGLAND, for painful skin tumors. Therefore, the authors also created a new acronym for painful tumors of the skin (LEND AN EGG) based upon the woman’s tumor, a reviewed of a dermatopathology textbook for lesions listed as either painful or tender, and their clinical experience; their mnemonic remained unchanged for 25 years.	[2]
Bhat, et al.^a^	2019	The authors modified the acronym by Naversen et al. based upon a 31-year-old man with three painful acquired tufted angiomas on the left side of his chest [7] and their awareness of the nocturnal pain associated with the cutaneous venous malformations of blue rubber bleb syndrome. They introduced the acronym BLEND TAN EGG.	[3]
Cohen, et al.	2019	The authors encountered two women with painful skin tumors that were not listed in the prior acronym: a 53-year-old with an organizing thrombus that presented as a tender firm nodule on her lateral left foot and a 56-year-old whose osteoma cutis lesion presented as a tender nodule on her left lower abdomen. After an extensive review of the literature, combined with their clinical experience, the authors discovered 25 painful skin tumors and introduced a new acrostic for painful tumors of the skin based upon the children’s book Charlotte’s Web [8]: CALM HOG FLED PEN AND GETS BACK.	[4]
Cohen, et al.	2020	The authors evaluated a 74-year-old man with a painful lesion of several years duration on his right leg; 15 years earlier, hot solder had dripped onto the site and become embedded beneath the skin. Since foreign body (solder) and the cutaneous reaction to the foreign body were not included in their recently introduced acrostic, the authors modified it by moving an ‘S’ (from GETS to create HOGS) and adding an ‘S’ (changing PEN to PENS) in order to add the new diagnosis of ‘something else’ (which would include not only foreign body and foreign body reaction, but also allow for additional painful tumors in the future). Their new mnemonic became CALM HOGS FLED PENS AND GET BACK.	[5]
Sargent, et al.	2022	The authors assessed a 70-year-old woman with a tender, slowly enlarging, blue-tinged subcutaneous nodule on her right proximal dorsal forearm for several months; an excisional biopsy revealed an intravascular lobular capillary hemangioma. The authors modified Cohen et al.’s mnemonic by removing the ‘S’ (something else) from PENS and adding the word ‘IF’ to include intravascular lobular capillary hemangioma and foreign body (reaction): IF HOGS FLED PEN, CALM AND GET BACK. However, the authors also emphasized that “future additions to the cutaneous tumor differential diagnosis may require creative additions and rearrangements to this acronym.”	[6]
Cohen	2022	The author presents the features of three women with painful dermatofibromas. The author also adds not only another painful skin tumor (lymphoma of the skin including B-cell lymphomas, T-cell lymphomas, and Hodgkin lymphoma), but also an ‘everything else’ category for the inclusion of future painful cutaneous neoplasms. Hence, the word ‘IF’ from Sargent et al.’s acronym is changed to ‘LIFE’ and the words are more appropriately arranged to create a new acrostic for painful tumors of the skin: HOG FLED PEN AND GETS CALM LIFE BACK.	CR

Several clinical and pathologic variants of dermatofibroma have been described [9,10]. A dermatofibroma commonly presents as a hyperpigmented dermal papule or nodule; however, the lesion can be hypopigmented to pink and it can be atrophic or depressed below the surrounding epithelium. A distinctive clinical characteristic of a dermatofibroma, the dimple sign, can be elicited when an individual uses their thumb and index finger to pinch the skin adjacent to the lesion; depression of the tumor and the surrounding skin, creating a dimple, is observed [2-4]. 

Microscopic examination of a dermatofibroma shows a dermal tumor consisting of benign fibroblasts and/or histiocytes. Compression and thickening of the collagen at the periphery of the tumor may be present creating keloidal-appearing collagen bundles. The overlying epidermis may demonstrate hyperkeratosis (morphologically appearing scaly), acanthosis (appearing as thickening of the epithelium), and basal layer hyperpigmentation (accounting for the darker color of the lesion clinically). In addition, some of the tumors have a Grenz zone of normal-appearing papillary dermis between the epidermis above and the dermal neoplasm below [9,10].

Dermatofibroma researchers have observed that some of the tumors were symptomatic. In a retrospective study of 122 biopsy-confirmed specimens of dermatofibromas from 92 patients, nearly 60% of the lesions were asymptomatic; however, 20.5% (25 tumors) were tender, 11.5% (14 tumors) were painful, and 9.8% (12 tumors) were pruritic [9]. Another study evaluated 75 tumors (including 72 dermatofibromas and three dermatomyofibromas) from 70 patients, between six months to 18 years old, and noted that the neoplasm was either painful or hypersensitive to touch in 27% (19 of 70) of the children [10]. Although the pathogenesis of dermatofibroma-associated pain remains to be established, one group of investigators has speculated that dermatofibroma-related pain develops as a result of entrapping the collagen bundles by the adjacent interlacing tumor strands of fibroblasts or histiocytes [3].

Aneurysmal dermatofibroma is a unique variant of this neoplasm with distinctive clinical presentation and pathologic features. Morphologically, they can be larger, cystic, and appear blue, black, or dark red; in addition, they can be associated with rapid growth and pain. Histologically, in addition to fibrous solid areas, up to half of the tumor consists of large, blood-filled, histiocyte-lined tissue spaces containing hemosiderin pigment, fibroblasts, and foam cells [11,12].

Cruz and Kyriakos are credited for the initial description of aneurysmal dermatofibroma. Their study included 17 cases from 14 women and three men. The lesion was described as painful in five (29%) of the patients [11]. Subsequently, Hoyt et al. described the features of a painful, biopsy-confirmed, aneurysmal dermatofibroma of three to four years duration on the left anteromedial thigh of a 30-year-old man. The lesion appeared as a moderately firm, violaceous 10 x 15-millimeter nodule beneath and surrounded by a pink scaly 3 x 2-centimeter patch with peripheral hyperpigmentation. Physical exertion would exacerbate the pain; however, oral administration of over-the-counter analgesics and topical application of ice packs would provide relief of the pain [12]. 

Atrophic dermatofibroma is another variant of dermatofibroma with characteristic morphologic and histologic features. Clinically, the tumor presents as a solitary patch with a central umbilication; it is most commonly found on either the shoulder, leg, or back of women over age 48 years. Pathologically, it has similar changes observed in non-atrophic dermatofibromas; however, in the central atrophic portion of the tumor, the dermis is often at least 50% thinned and the elastic fibers are either decreased or absent in this area [13].

Atrophic dermatofibroma has been reported in at least 102 individuals; two of these men had tumors that were transiently painful and demonstrated not only atrophic changes but also aneurysmal histologic characteristics. The first patient was a 27-year-old man who presented with an asymptomatic brown atrophic patch of two years duration on his back that had been temporarily painful and swollen one year earlier; a biopsy of the tumor was diagnosed as an aneurysmal dermatofibroma with atrophic features and the residual neoplasm was completely excised. The second patient was a 40-year-old man who had an asymptomatic nodular lesion of ten years duration on his left upper trunk that had become painful during the previous 12 hours; examination showed a five-centimeter hemorrhagic plaque that evolved into a hematoma during the subsequent week and then progressed into an asymptomatic depressed lesion two months later. The lesion was completely excised and demonstrated an atrophic dermatofibroma in the atrophic zone accompanied by aneurysmatic changes in the non-atrophic area [13].

Single case reports of painful tumors in individuals with other variants of dermatofibroma have also been described. A 25-year-old woman with a deep penetrating dermatofibroma of her right foot presented with a slowly growing painful mass of two months duration that made walking difficult; the tumor was completely excised and the dermatofibroma-associated pain gradually subsided [14]. Another patient, a 19-year-old woman, presented with a 2.1 x 1.5 x 1.0-centimeter nodule on her medial left thigh that was tender to touch; a diagnosis of subcutaneous dermatofibroma was established after evaluation of the completely excised tumor [15].

Three patients, each with a painful dermatofibroma, are described in this report; their characteristics are summarized in Table 3. The women ranged in age from 35 to 42 years (median, 37 years); one of the women was a liver transplant recipient and was receiving immunosuppressive therapy. Lesion-associated pain had been present for several months to at least a year (median, approximately one year). The benign tumors presented as flesh-colored to slightly red or brown or tan dermal nodules (with or without surrounding hyperpigmentation) that ranged in size from 5 x 5 millimeters to 12 x 12 millimeters (median, 8 x 8 millimeters) and were located on either the buttock or shoulder or arm. Two women had a classic (fibrocollagenous) dermatofibroma and the third woman had a histiocytic dermatofibroma. Pain resolved after the biopsy and did not recur even though there was microscopic evidence of residual tumor following the biopsy of the lesions.

**Table 3:** Characteristics of patients with a painful dermatofibroma

Features	Patient one	Patient two	Patient three
Age	42-year-old	37-year-old	35-year-old
Race	Philippine	Hispanic	Caucasian
Gender	Woman	Woman	Woman
Medical history	None	Liver transplant, migraine headaches	Healthy
Dermatology history	None	Junctional nevi, plantar verruca	None
Skin cancer history	None	None	None
Duration	Approximately one year	Several months	At least 12 months
Location	Left buttock	Left shoulder	Left arm
Symptoms	Pain	Pain	Pain, pruritus
Morphology	Protuberant, brown dermal nodule surrounded by a hyperpigmented patch	Slighty raised, tan dermal nodule	Prominent, tender, flesh-colored to slightly red dermal nodule
Size	12 x 12-millimeters nodule within 3 x 2-centimeter patch	5 x 5-millimeters	8 x 8-millimeters
Dermatofibroma variant	Classic (fibrocollagenous)	Classic (fibrocollagenous)	Histiocytic
Treatment	Incomplete removal by punch biopsy followed by observation	Incomplete removal by punch biopsy followed by observation	Incomplete removal by punch biopsy followed by observation
Follow-up	Pain resolved without recurrence	Pain resolved without recurrence	Pain resolved without recurrence

In addition to dermatofibroma, there are many other tumors of the skin that are painful. Mnemonic devices have been used as memory aids to facilitate remembering the differential diagnosis of painful skin tumors (Table 1) [1-6]. As additional cutaneous conditions were discovered to be painful, new acronyms and acrostics were created to accommodate the diagnoses (Table 2) [1-8].

Earlier acronyms for painful skin tumors were limited to seven, nine, or 11 conditions [2,3]. However, the differential diagnosis eventually expanded to 25 conditions [4]. Indeed, based on the extensive differential diagnosis of tender cutaneous neoplasms, investigators created a unique acrostic that was inspired by E. B. White’s children’s book Charlotte’s Web [8].

When these investigators subsequently encountered an individual with a painful skin lesion caused by a foreign body (solder) and the cutaneous reaction to that foreign body, they modified their original acrostic by adding a “something else” category [5]. Yet, within two years, other researchers not only removed the new category, but also added two more conditions -- foreign body (reaction) and intravascular lobular capillary hemangioma -- and also changed the acrostic [6]. However, these researchers also emphasized that “future additions to the cutaneous tumor differential diagnosis may require creative additions and rearrangements to this acronym” [6].

Since the most recently proposed acrostic, a thorough review of the literature has revealed another painful tumor of the skin: lymphoma. Shelley and Wood observed that pain was a prominent symptom associated with occult malignant lymphomas in the skin of 50% (three of six) of their patients [16]. Subsequently, painful cutaneous lymphomas have been reported in patients with primary cutaneous B-cell lymphoma, subcutaneous panniculitis-like T-cell lymphoma, mycosis fungoides (tumor stage), and primary cutaneous Hodgkin lymphoma [17-20].

Thus, lymphoma of the skin should be added to the list of differential diagnoses for tender cutaneous neoplasms. However, in an attempt to maintain the integrity of the acrostic for painful skin tumors, it is also reasonable to add an additional category (which would be designated “everything else”) for new painful cutaneous lesions that are subsequently discovered. Hence, after modification and word rearrangement of the most recent mnemonic device, a new acrostic for painful skin tumors that still maintains its inspiration from Charlotte’s Web is proposed: HOG FLED PEN AND GETS CALM LIFE BACK (Table 1) [1-6].

## Conclusions

There are several benign and malignant tumors that can present as tender cutaneous neoplasms. Dermatofibroma is a commonly occurring benign tumor that usually appears as an asymptomatic dermal papule or nodule. However, approximately 30% of dermatofibromas have presented as a painful or tender lesions. Indeed, dermatofibromas are the most common diagnosis associated with a painful tumor of the skin. The characteristics of three women with symptomatic dermatofibromas are reported; a punch biopsy, which removed some but not all, of the painful dermal nodule, not only established the diagnosis of the neoplasm but also resulted in resolution of the dermatofibroma-associated pain without recurrence. In addition to dermatofibromas and other fibrous lesions, tender cutaneous neoplasms include tumors of adipose, bone, calcium, cartilage, eccrine, lymphoproliferative, infiltrative, muscle, neural, and vascular origin. Mnemonic devices have been created to aid clinicians in remembering the differential diagnosis of painful skin tumors. Shorter acronyms (such as GLENDA, ENGLAND, LEND AN EGG, and BLEND TAN EGG) were adequate when the number of possible pain-related cutaneous conditions was 11 or less. When the differential diagnosis of tender cutaneous neoplasms expanded to 25 tumors, an acrostic inspired by E. B. White’s children’s book Charlotte’s Web was created; subsequently, two minor revisions to the mnemonic were published for each additional tender skin lesion that was identified. With the addition of cutaneous lymphoma and a final category of “everything else” to maintain the integrity of mnemonic, a new acrostic for the painful tumors of the skin -- that is still able to incorporate the inspiration from Charlotte’s Web -- has been proposed: HOG FLED PEN AND GETS CALM LIFE BACK.
